# A method to evaluate body length of live aquatic vertebrates using digital images

**DOI:** 10.1002/ece3.7444

**Published:** 2021-04-05

**Authors:** Ivan Arismendi, Gwen Bury, Lauren Zatkos, Jeff Snyder, David Lindley

**Affiliations:** ^1^ Department of Fisheries and Wildlife Oregon State University Corvallis USA; ^2^ Oak Ridge Institute for Science and Technology Fellowship hosted by USDA Forest Service PNW Research Station Corvallis USA; ^3^ Department of Biology Western Oregon University Monmouth USA; ^4^ L.N. Curtis & Sons Walnut Creek USA

**Keywords:** experimental forest, Long‐term Ecological Research, salamander, stream network, synoptic sampling, trout

## Abstract

Traditional methods to measure body lengths of aquatic vertebrates rely on anesthetics, and extended handling times. These procedures can increase stress, potentially affecting the animal's welfare after its release. We developed a simple procedure using digital images to estimate body lengths of coastal cutthroat trout (*Oncorhynchus clarkii clarkii*) and larval coastal giant salamander (*Dicamptodon tenebrosus*). Images were postprocessed using ImageJ2. We measured more than 900 individuals of these two species from 200 pool habitats along 9.6 river kilometers. The percent error (mean ± SE) of our approach compared to the use of a traditional graded measuring board was relatively small for all length metrics of the two species. Total length of trout was −2.2% ± 1.0. Snout–vent length and total length of larval salamanders was 3.5% ± 3.3 and −0.6% ± 1.7, respectively. We cross‐validated our results by two independent observers that followed our protocol to measure the same animals and found no significant differences (*p* > .7) in body size distributions for all length metrics of the two species. Our procedure provides reliable information of body size reducing stress and handling time in the field. The method is transferable across taxa and the inclusion of multiple animals per image increases sampling efficiency with stored images that can be reviewed multiple times. This practical tool can improve data collection of animal size over large sampling efforts and broad spatiotemporal contexts.

## Graphical abstract

We developed a procedure to estimate body length of aquatic vertebrates using digital images of live animals in the field and post processing using ImageJ2. This method is reliable and reduces stress and handling time in the field. This tool can improve our understanding about species responses to disturbances over large sampling efforts and broader spatiotemporal contexts.

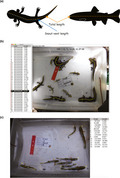

## 1 INTRODUCTION

The role of body size in shaping the form and function of animals has attracted the attention of scientists for decades (Calder, 1996). Larger body size provides crucial benefits including higher fitness and fecundity (Barneche et al., 2018), and competitive advantages within (Newman, 1956) and among species (Persson, 1985). A reduction in body size has been proposed as the third universal response of animals to the warming climate (Daufresne et al., 2009). Yet, empirical evidence to test this hypothesis at broader spatiotemporal contexts is lacking in freshwater ecosystems.

Traditional methods of measuring fish and amphibians often involve use of anesthetics and prolonged handling (Bonar et al. 2009, Bury and Corn 1991, Stetter, 2001), which can cause stress (Bliley and Woodley 2012, Carter et al., 2011) and have negative postrelease effects on animals. Recent developments of inexpensive water‐resistant, high‐resolution digital cameras with large storage capacity, coupled with open‐source image processing software could be used to estimate body size. Digital images have been used to estimate growth rates of *Rana sylvatica* tadpoles (Davis et al., 2008), body size of the marbled salamander *Ambystoma opacum* (Mott et al., 2010), and body length of coral reef fishes (Andrialovanirina et al., 2020). However, the use of digital images to estimate body size of live aquatic vertebrates from large‐scale sampling efforts is limited. We present a practical tool, without the use of anesthetics to estimate body size from live animals based on digital images taken in the field and posterior image processing in the lab.

### 1.1 Description and implementation

Our method is compatible with survey procedures that capture live animals (e.g., electrofishing, traps, seine nets). We used a single‐pass backpack electrofishing technique for wadable streams without blocknets for riverscape‐scale studies (Foley et al., 2015; Matson et al., 2018). We sampled coastal cutthroat trout (*Oncorhynchus clarkii clarkii*) and larval coastal giant salamander (*Dicamptodon tenebrosus*) from 200 pool habitats along 9.6 km of the mainstem of Lookout Creek at the H.J. Andrews Experimental Forest, Oregon from August 26–30, 2019. At each sampled pool, all shocked animals were captured using hand nets and transferred to buckets of aerated stream water.

**FIGURE 1:**
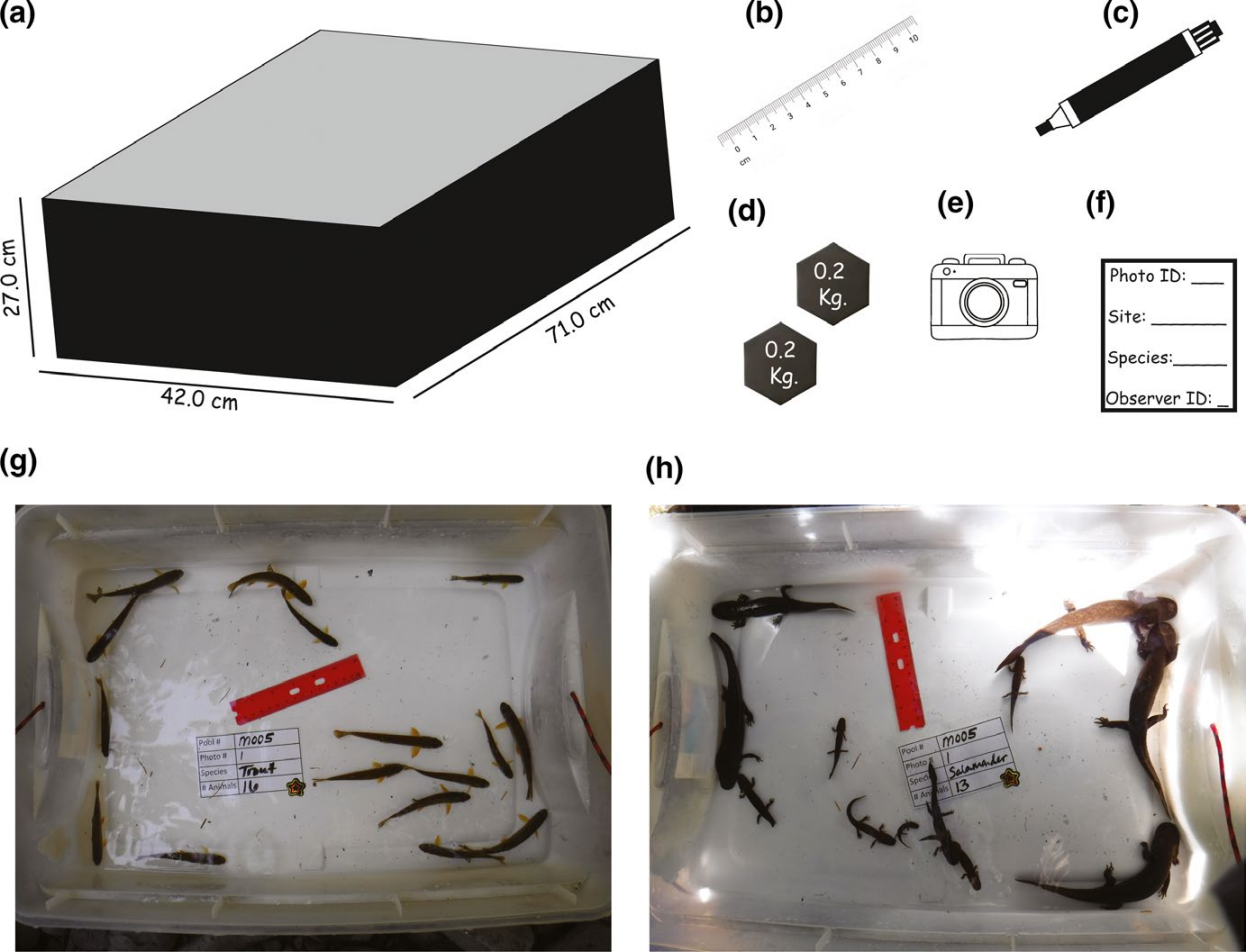
The list of supplies needed to conduct body length surveys of live aquatic vertebrates included a white plastic container (a), ruler to be used as reference scale (b), dry erase marker (c), weighted units to keep ruler and writing slate at the bottom of the container if needed (d), digital camera (e), and underwater writing slate (f). Examples of images under real field conditions including larval coastal giant salamander (g) and coastal cutthroat trout (h)

We separated animals by species and transferred them to a plastic container in groups of 1–20 individuals (Figure 1). The plastic container dimensions can be selected based on needs of transportability and access to field sites, whereas the color of the container must ensure enough contrast between target species and background. We kept minimal stream water levels in the container (5–8 cm water depth) to maintain all animals at the same depth, thereby minimizing potential image distortion. Each group of animals was photographed from a distance between 50 and 80 cm based on site field conditions. For each photograph, we assigned a unique identification number labeled in an underwater writing slate placed at the bottom of the container next to a reference ruler (Figure 1). We used rocks as weights to keep both the ruler and writing slate at the bottom of the container. We took between 1 and 5 photographs of each setting using a digital camera (Fujifilm model FinePix XP 130). Multiple photographs allowed for maximum visibility of target animals, the reference ruler, and the unique identification label. After the animals were photographed, they were released to the pool in which they were captured. The duration of our digital photographing procedure ranged between 2 and 5 min per pool habitat.

**FIGURE 2:**
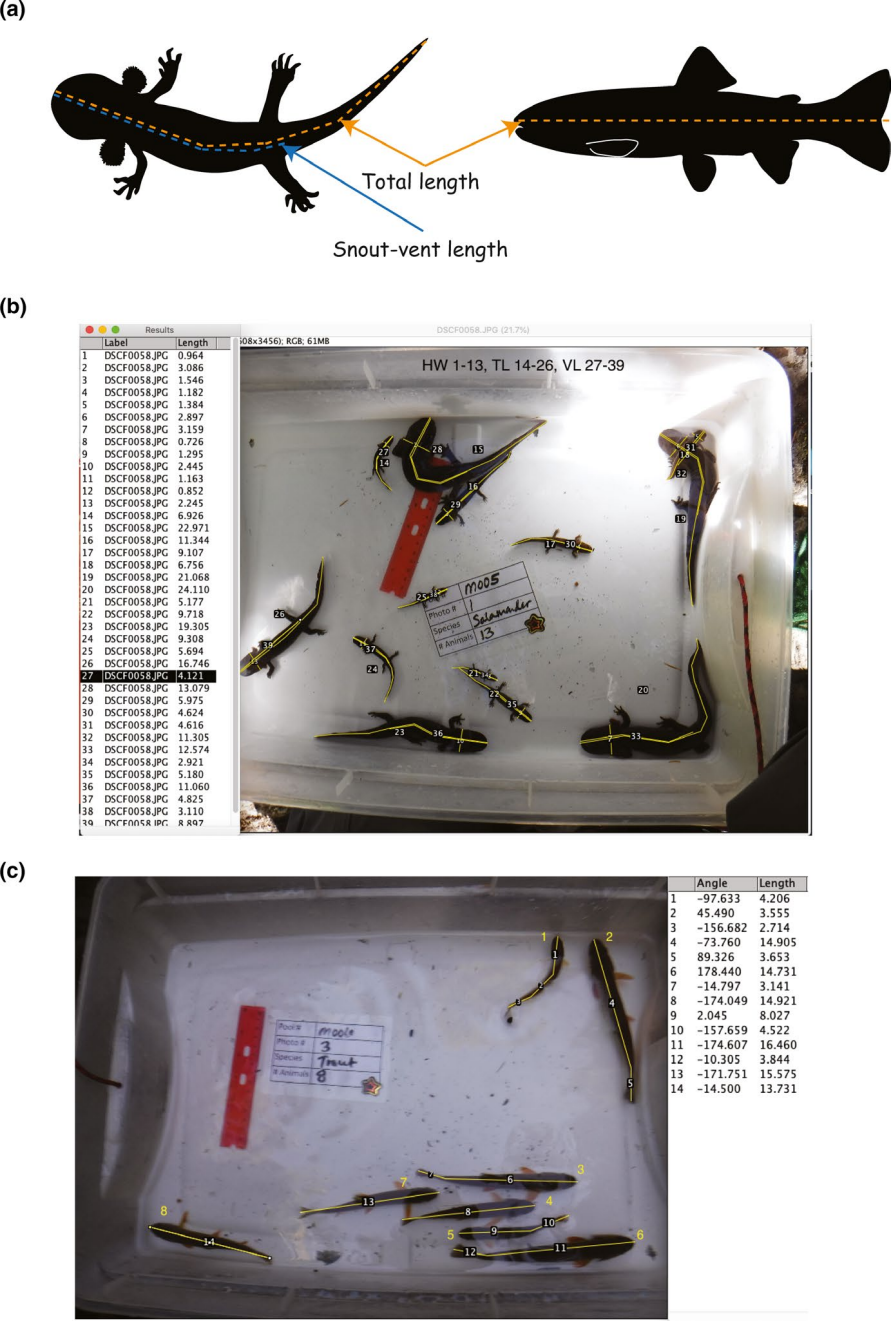
Body size estimates for each species (a) and examples of screenshots of larval coastal giant salamander (b) and coastal cutthroat trout (c) following our protocol in ImageJ2. When animals were curved, we used multiple segments that were added to estimate body length

We used 17 larval salamanders and 47 trout to contrast our approach with the traditional use of a graded measuring board. We transferred animals to different buckets containing buffered tricaine methanesulfonate (MS‐222) as an anesthetic. We used 2.5 ml buffered MS‐222L solution from stock solution of 20g MS222/L, and in a different bucket, we duplicated the dose for larval salamanders. We kept animals in anesthetic until major locomotion ceased. We measured each animal to the nearest millimeter using a graded measuring board and then placed measured animals in a recovery bucket containing aerated stream water. We measured snout–vent and total lengths for larval salamanders, whereas we measured total length for trout (Figure 2). After recovering, all animals were released into the pool in which they were captured.

### 1.2 Image processing using imageJ2

We processed all images in ImageJ2 (Schindelin et al., 2015). The protocol below includes instructions to open images and prepare them for measurements. The observer could adjust the contrast and/or brightness of images to facilitate the visualization of target objects and set parameters for calibration, measurement and storage.

#### 1.2.1 Step 1. Image calibration in imageJ

We performed a calibration on each image before processing and verified that the reference ruler was present in each of them (Figure 2). We selected the straight‐line measuring tool on the ImageJ task bar and measured the distance of 10 mm between any two, clear visible points on the reference ruler. We chose the “analyze” option on the task bar followed by “set scale” on the drop down to set the scale of the image per pixel. Observers can choose other units of measurement (i.e., inches, centimeters) if needed. We then closed the set scale dialog.

#### 1.2.2 Step 2. Measuring in imageJ

After the image calibration was completed, we took most measurements using the segmented line tool, as most animals were curved. To use the segmented line tool, we clicked once on the left button of the mouse and performed single click increments following the curve of the animal and double‐clicked when the measurement was completed. We then pressed “T” on the keyboard to bring up the Region of Interest (ROI) manager and to record the animal size measurement. The ROI function allowed us to make multiple measurements, label and show them for each individual animal (Figure 2). We displayed measurements by number in the dialog box associated with the number labeled in the image we were processing.

#### 1.2.3 Step 3. Data storage

We arranged the image and results dialog box to be visible on the screen so as not to cover any labels, ruler, or animals. We took a screen shot and used Microsoft Paint software to paste the image and create a text box that allowed us to label the numbers within the image with their associated animals and measurements. For example, if we measure thirteen animals, had thirty‐nine measurements and knew we started with a measurement of head width (HW) followed by total length (TL) and then snout–vent length (SV), we labeled the image with a text box reading “1–13 HW, 14–26 Tl, 27–39 SV” (Figure 2). We saved the image for future verification. The image processing took approximately 4 min per image and 30 s per body length estimate. Duration time included the calibration, length estimate, and data storage. Image processing time varied based on image clarity, number of animals in the image, animal orientation, and the number of length estimate attempts.

### 1.3 Accuracy and cross‐validation

**FIGURE 3:**
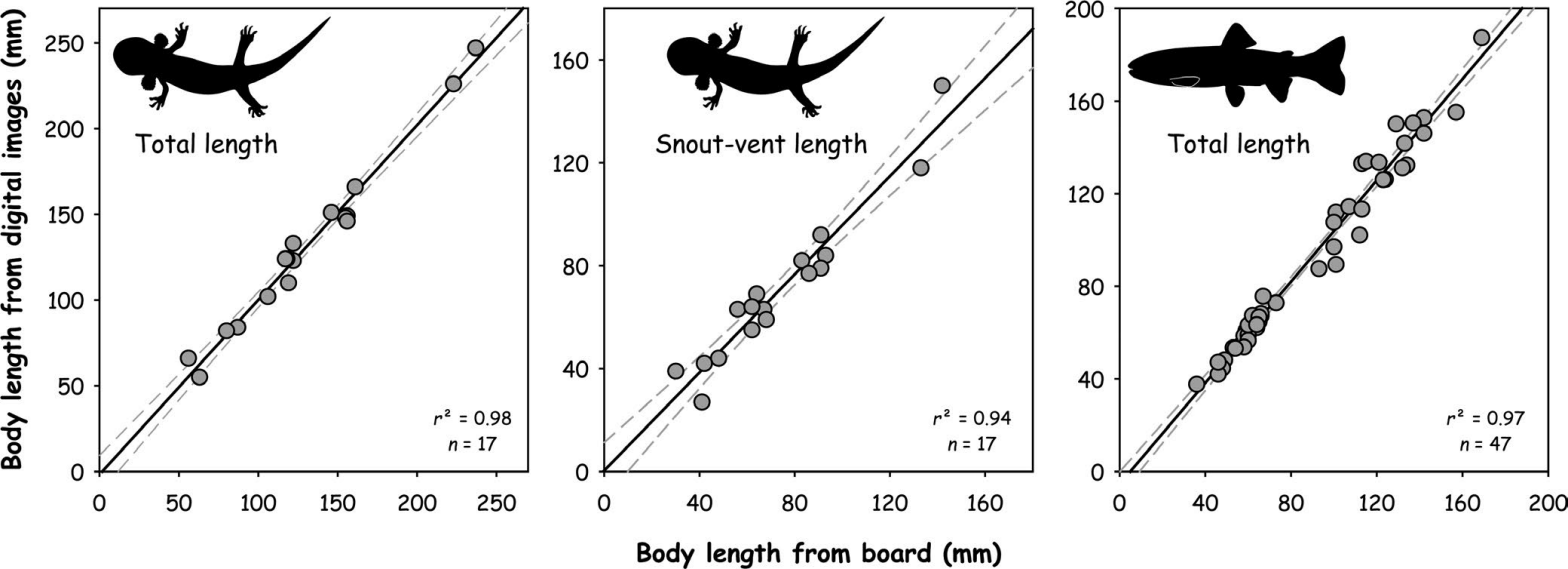
Relationship between paired observations of body length from the graded measuring board versus digital images for total length (left) and snout–vent length (middle) of larval coastal giant salamander, and total length of costal cutthroat trout (right)

We used a simple linear regression to compare paired observations of body length measurements obtained from a graded measuring board with body length estimations from digital images (Figure 3). We found a statistically significant positive relationship (*p* <.001) between body length paired observations with slope estimates close to 1 in all cases. For total length of larval coastal giant salamander, the mean slope was 1.02 ± 0.04 SE and for snout–vent length it was 0.95 ± 0.06 SE. For total length of coastal cutthroat trout, the mean slope was 1.09 ± 0.03 SE. In addition, we used a two‐tailed Mann–Whitney *U* statistic with Yates continuity correction to test for potential differences in medians between body length observations from the graded measuring board and body length estimates. We adopted this nonparametric test after failing the assumption of data normality. We found no significant differences between groups for total length (Mann–Whitney *U* = 140.5, *n*_1_ = *n*_2_ = 17, *p* =.904) and snout–vent length (*U* = 134, *n*_1_ = *n*_2_ = 17, *p* =.730) of larval coastal giant salamander, or for total length of coastal cutthroat trout (*U* = 1,057.5, *n*_1_ = *n*_2_ = 47, *p* =.725). Lastly, we estimated the percent error of our approach compared to the use of traditional graded measuring board using % error = [(*BL_board_*_‐_ *BL_image_*) / *BL_board_*] x 100 where *BL_board_* represented the body length of the individual measured from a graded measuring board and *BL_image_* represented the body length of the individual estimated from a digital image. For larval coastal giant salamander (*n* = 17), the percent error (mean ± SE) for snout–vent length and total length was relatively small (i.e., 3.5% ± 3.3 and −0.6% ± 1.7 respectively). Similarly, the percent error (mean ± SE) for total length of coastal cutthroat trout (*n* = 47) was small (i.e., −2.2% ± 1.0). The percent error of our body length estimates can be influenced by the parallax on distortion effect, which is when the measurement of body length is more or less than the true length because the camera was positioned at an oblique angle with respect to the ruler. We reduced the parallax on distortion effect by consistently orienting our line of sight directly above the ruler and maintaining low water levels in the container (5–8 cm) to avoid locating animals at multiple water depths.

**FIGURE 4:**
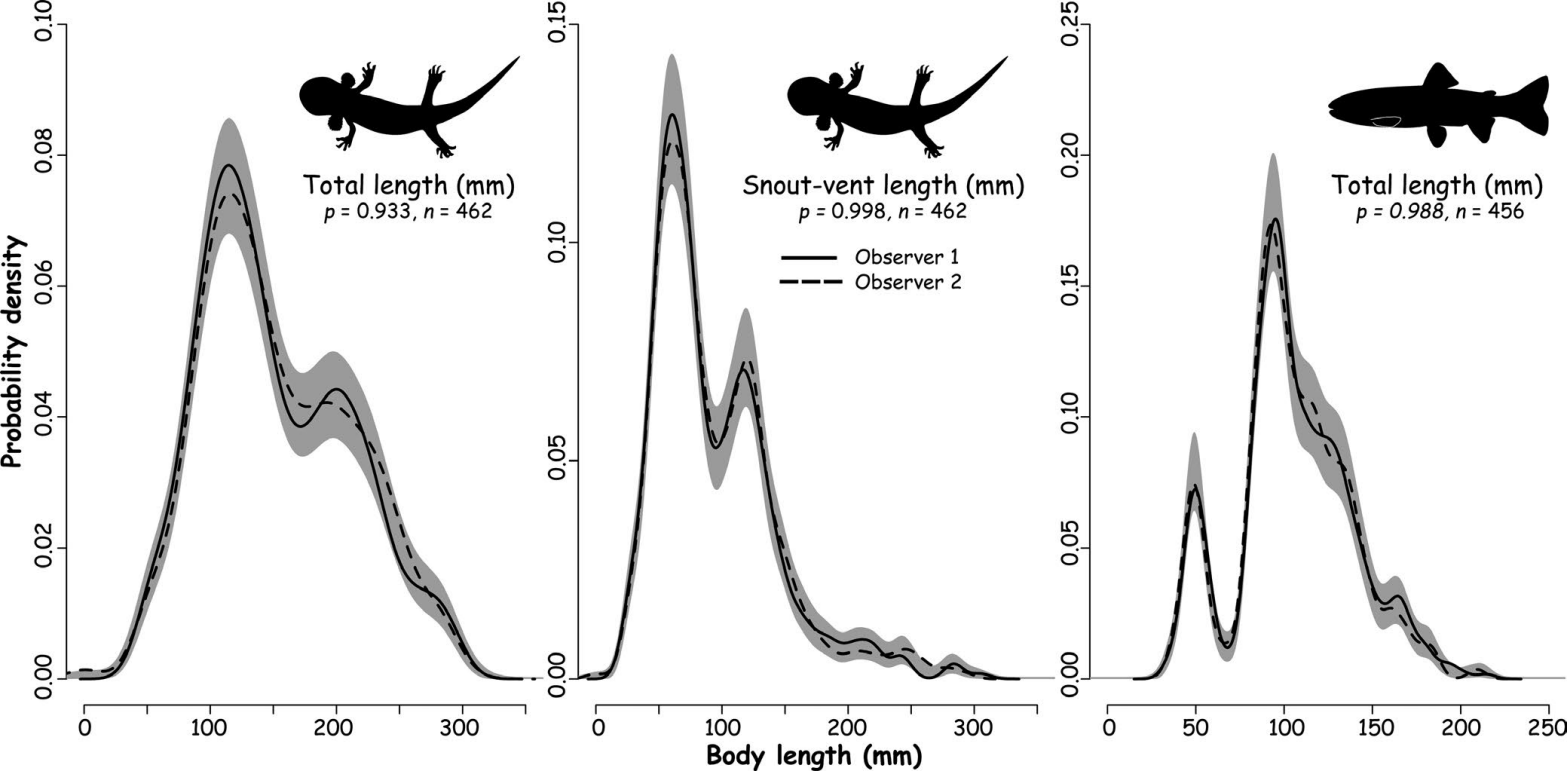
Body length (total length and snout–vent length) distributions of aquatic vertebrates including larval coastal giant salamander (left and middle panel) and costal cutthroat trout (right panel). Body length of individual animals was extracted from digital images using two independent observers (solid and dotted lines respectively). The gray‐shaded area represents ± 1 SE of the null model of no difference in length measurements between observers

We cross‐validated our approach by using two independent observers following our protocol. They estimated the lengths of all animals from 268 digital images. These included 200 images that included 462 larval coastal giant salamanders, and 68 images that included 456 coastal cutthroat trout. The number of larval salamanders per image ranged between 1 and 16 with a mean of 3 ± 0.3 SE whereas the number of trout per image ranged between 1 and 22 with a mean of 11 ± 1.0 SE. We used kernel density estimates to compare body size distributions between the two independent observers (Langlois et al., 2012). Specifically, we used the “dpik” function and Sheather–Jones method to select bandwidths (Sheather & Jones, 1991) included in the package “KernSmooth” (Wand, 2013) in R (R Core Team, 2013). We used the permutational “sm.density.compare” function (99,999 permutations) in the package “sm” (Bowman & Azzalini, 2014) to test for differences in body size distributions between observers. The “sm.density.compare” function randomly assigned body size values between the two observers and estimated how different the observed data were from the null hypothesis using a randomization procedure along the length distribution. We found no evidence of differences in body size distributions between the two independent observers for the total length and snout–vent for larval salamanders and total length for trout (Figure 4).

## 2 CONCLUSION

We present an accurate procedure to estimate body size data of live aquatic vertebrates in the field. Our proposed method is reliable and reduces direct animal handling. This practical tool can be used on an array of vertebrates where body size data are needed. Additional morphometric measurements (i.e., head architecture of larval salamanders) can be obtained, documented, and analyzed with stored images.

## CONFLICT OF INTEREST

The authors declare no conflict of interest associated with this manuscript.

## AUTHOR CONTRIBUTION

**Ivan Arismendi:** Conceptualization (lead); Data curation (equal); Formal analysis (lead); Funding acquisition (lead); Investigation (lead); Methodology (lead); Project administration (lead); Resources (lead); Software (equal); Supervision (equal); Validation (equal); Visualization (lead); Writing‐original draft (lead); Writing‐review & editing (lead). **Gwen Bury:** Conceptualization (supporting); Data curation (equal); Formal analysis (supporting); Methodology (equal); Project administration (supporting); Supervision (equal); Writing‐review & editing (supporting). **Lauren Zatkos:** Data curation (equal); Methodology (supporting); Project administration (equal); Supervision (equal); Validation (equal); Writing‐review & editing (supporting). **Jeff Snyder:** Conceptualization (supporting); Funding acquisition (equal); Investigation (supporting); Methodology (supporting); Project administration (supporting); Resources (supporting); Writing‐review & editing (supporting). **David Lindley:** Data curation (equal); Methodology (supporting); Validation (equal); Writing‐review & editing (supporting).

## Data Availability

The data that support the findings of this study have been deposited in Dryad. https://doi.org/10.5061/dryad.34tmpg4js
